# Relationships between coagulation factors and thrombin generation in a general population with arterial and venous disease background

**DOI:** 10.1186/s12959-022-00392-0

**Published:** 2022-06-08

**Authors:** Pauline C. S. van Paridon, Marina Panova-Noeva, Rene van Oerle, Andreas Schulz, Jürgen H. Prochaska, Natalie Arnold, Irene Schmidtmann, Manfred Beutel, Norbert Pfeiffer, Thomas Münzel, Karl J. Lackner, Hugo ten Cate, Philipp S. Wild, Henri M. H. Spronk

**Affiliations:** 1 Laboratory for Clinical Thrombosis and Hemostasis, Department of Internal Medicine, Cardiovascular Research Institute Maastricht (CARIM), Maastricht University Medical Center, Maastricht, 6200 MD the Netherlands; 2 Center for Thrombosis and Hemostasis (CTH), University Medical Center of the Johannes Gutenberg-University Mainz, Mainz, Germany; 3 DZHK (German Center for Cardiovascular Research), Partner Site RhineMain, Mainz, Germany; 4 Preventive Cardiology and Preventive Medicine, Center for Cardiology, University Medical Center of the Johannes Gutenberg-University Mainz, Mainz, Germany; 5 Institute of Medical Biostatistics, Epidemiology and Informatics, University Medical Center of the Johannes Gutenberg-University Mainz, Mainz, Germany; 6 Department of Psychosomatic Medicine and Psychotherapy, University Medical Center of the Johannes Gutenberg-University Mainz, Mainz, Germany; 7 Department of Ophthalmology, University Medical Center of the Johannes Gutenberg-University Mainz, Mainz, Germany; 8 Center for Cardiology I, University Medical Center of the Johannes Gutenberg-University Mainz, Mainz, Germany; 9 Institute for Clinical Chemistry and Laboratory Medicine, University Medical Center of the Johannes Gutenberg-University Mainz, Mainz, Germany

**Keywords:** Coagulation factors, Thrombin generation, Arterial thrombosis, Venous thrombosis

## Abstract

**Background:**

The current study aims to identify the relationships between coagulation factors and plasma thrombin generation in a large population-based study by comparing individuals with a history of arterial or venous thrombosis to cardiovascular healthy individuals.

**Methods:**

This study comprised 502 individuals with a history of arterial disease, 195 with history of venous thrombosis and 1402 cardiovascular healthy individuals (reference group) from the population-based Gutenberg Health Study (GHS). Calibrated Automated Thrombography was assessed and coagulation factors were measured by means of BCS XP Systems. To assess the biochemical determinants of TG variables, a multiple linear regression analysis, adjusted for age, sex and antithrombotic therapy, was conducted.

**Results:**

The lag time, the time to form the first thrombin, was mainly positively associated with the natural coagulant and anti-coagulant factors in the reference group, i.e. higher factors result in a longer lag time. The same determinants were negative for individuals with a history of arterial or venous thrombosis, with a 10 times higher effect size. Endogenous thrombin potential, or area under the curve, was predominantly positively determined by factor II, VIII, X and IX in all groups. However, the effect sizes of the reported associations were 4 times higher for the arterial and venous disease groups in comparison to the reference group.

**Conclusion:**

This large-scale analysis demonstrated a stronger effect of the coagulant and natural anti-coagulant factors on the thrombin potential in individuals with a history of arterial or venous thrombosis as compared to healthy individuals, which implicates sustained alterations in the plasma coagulome in subjects with a history of thrombotic vascular disease, despite intake of antithrombotic therapy.

**Supplementary Information:**

The online version contains supplementary material available at 10.1186/s12959-022-00392-0.

## Introduction

Thrombin generation (TG) is established as an important research tool for exploring the plasma “coagulome” in relation to clinical risks for bleeding or thromboembolism. For a bleeding tendency, like hemophilia subjects lacking factor VIII or IX, reduced peak height and endogenous thrombin potential (ETP) of the TG curve have been observed, supporting a state of hypocoagulability [1–5]. Correction of such factor deficiency normalized the TG profile [6]. In thrombosis research, the reported findings on TG are conflicting, e.g. whereas an increased thrombin potential is frequently reported in venous thrombosis, for subjects with arterial thrombosis data are quite inconsistent [7–10].While some studies show positive associations of increased peak height and/or ETP to outcomes like ischemic stroke, other studies show reverse associations of increased lag time and/or lower peak height levels in patients that suffered a myocardial infarction or stroke [11, 12]. The reasons for these discrepancies are not fully understood but might include variations in coagulation factor concentrations, release of tissue factor pathway inhibitor from the endothelium as well as effects of specific medication. Solid evidence based on a comprehensive set of different data is still missing [13].

Venous thromboembolism (VTE) and arterial thrombotic diseases share several risk factors and several studies have shown that the risk of arterial thrombosis is increased in those that suffered a first VTE and vice versa [14]. Therefore, one would expect that also the plasma coagulome, assessed by the TG assay, would reflect certain similarities between subjects with VTE or arterial thrombosis [15]. However, given the observed discrepant associations with TG data, different profiles between venous and arterial thrombotic disease may also be present.

In order to address these issues, we carried out the present study to identify the relationships between (natural anti-) coagulation factors and parameters of the TG in individuals with a history of either an arterial cardiovascular disease or venous thrombotic disease compared to cardiovascular healthy group within the population-based Gutenberg Health Study.

## Methods

### Research design

The Gutenberg Health Study (GHS) is a prospective, observational, single center cohort study, designed for population-based health research, in the Rhine-Main region in Germany. With a total of 15,010 individuals between 35 and 74 years enrolled at the baseline examination, the GHS aims to assess the consequences of diseases and environmental factors in addition to the inherited predisposition on the development and progression of asymptomatic and symptomatic disease. During the baseline visit, every participant underwent a comprehensive, standardized 5-hour clinical examination program, as reported elsewhere [16, 17]. The baseline visit at the GHS study centre comprised a standardized 5-h investigation according to standard operating procedures. Participants underwent a detailed computer-assisted interview covering assessment of cardiovascular risk factors, lifestyle, socioeconomic status, and other areas. The prevalence of cardiovascular disease was determined by history taking. In addition to the extensive clinical assessment, a large biobank has been established for future biochemical and genetic analyses. As part of the follow-up, a standardized computer-assisted telephone interview and an inventory of primary and secondary endpoints were done 2.5 years after baseline visit. In addition, participants undergo a quinquennial, extensive clinical examination in the same research facility as the baseline visit. Primary endpoints of the study were myocardial infarction and cardiovascular death. Secondary endpoints were cerebrovascular accident, diabetes mellitus, heart failure, atrial fibrillation or death caused by the previously named diseases. Details of the study protocol and the further purposes of the study are discussed elsewhere [18].

### Study sample

From the initial GHS cohort including 5000 subjects TG data was available for 4843 subjects. From these, 1402 individuals were included in the reference group, 502 in the arterial disease group, and 195 in the venous disease group. Selection for each group was as follows:”

The overall study sample consisted of the first 5000 subjects enrolled into the GHS between April 2007 and October 2008. After excluding subjects without biomaterial available or without complete TG assessment (one or several TG parameters were missing), 4843 individuals were successfully included in the present analysis.

The reference group was defined as apparently cardiovascular healthy subjects without history of cardiovascular diseases (myocardial infarction [MI], congestive heart failure [CHF], coronary artery disease [CAD], peripheral artery disease [PAD], venous thromboembolism [VTE], atrial fibrillation [AF]), presence of cardiovascular risk factors (CVRF; obesity, dyslipidemia, arterial hypertension, diabetes mellitus) and included 1402 individuals. Individuals with a self-reported history of inherited coagulation abnormalities were excluded from the reference sample. The arterial disease group was defined as individuals with a history of MI, CAD, stroke or PAD and included 502 individuals. The venous disease group was defined as individuals with a history of deep venous thrombosis (DVT) or pulmonary embolism (PE) and included 195 individuals. Individuals that did not meet the above-mentioned criteria of the various groups were excluded from the current analysis. For a detailed definition of traditional CVRF and categorization of medications, please see Supplemental Material, Part A.

### Blood sampling and laboratory assessment

Venous blood sampling was performed according to standard operating procedures and the blood was collected in trisodium citrate (0.109 M, 1:9 vol:vol) monovette plastic tubes, while the subject was in fasting state (i.e. overnight fast, if subject was examined before 12 p.m.. and 5 hour fast, if subject was examined after 12 p.m.). Platelet poor plasma (PPP) was prepared by one-step centrifugation at 2000 x g at room temperature for 10 minutes. After preparation the PPP was aliquoted and immediately stored at − 80 °C in the Biobank of the GHS study center.

The TG was assessed in the Laboratory for Clinical Thrombosis and Hemostasis, Maastricht University, the Netherlands, by the Calibrated Automated Thrombogram (CAT) assay (Thrombinoscope BV, Maastricht, The Netherlands), according to the recommendations [19, 20]. The TG was triggered by PPP Reagent Low (Stago) in freshly thawed PPP. The CAT method employs a low affinity fluorogenic substrate for thrombin (Z-Gly-Gly-Arg-AMC) to continuously monitor thrombin activity in clotting plasma. TG measurements were calibrated against the fluorescence curve obtained in a sample from the same plasma (80 μL), supplemented with a fixed amount of thrombin–alfa 2-macroglobulin complex (20 μL of Thrombin Calibrator; Thrombinoscope BV, Maastricht, The Netherlands) and 20 μL of the fluorogenic substrate and calcium chloride mixture. TG parameters were derived from the TG curve and include lag time (time to minimum thrombin formed [min]), peak height (the maximum amount of thrombin formed [nM]) and endogenous thrombin potential (ETP or area under the curve [nM.min]).

Coagulation factors were measured by means of BCS XP Systems in the Biomolecular laboratory at the Department of Epidemiology, University Medical Center Mainz, Germany. The coagulation factors II, V, VII, VIII, IX, X, XI, XII were determined using the clotting-based coagulation methodology, protein C and antithrombin by the chromogenic assay and von Willebrand factor (vWF) and protein S by using immunological-based assay. Reference values by the WHO standard provided by Siemens were used. Total TFPI activity was assessed in PPP by the Actichrome TFPI activity assay (American Diagnostica, Stamford, CT, USA) in the Laboratory for Clinical Thrombosis and Hemostasis, Maastricht University, the Netherlands.

### Data management and statistical analysis

A central data management unit conducted quality control on all data in this study. Statistical analysis was performed with software program R, version 3.3.1 (http://www.R-project.org). Data on coagulation factors and inhibitors are presented as mean (standard deviation) in case of normal distribution.

Multiple linear regressions were used to assess the associations between biochemical variables and TG parameters in the reference group as well as in the arterial and venous disease group. The analyses were adjusted for age, sex and additionally for hormones (oral contraceptives and hormone replacement therapy = G03) and anti-coagulant agents (B01AA, B01AB, B01AE, B01AF, B01AX) as these may affect the thrombin potential. Due to a skewed distribution, lag time, as a dependent variable, was log-transformed prior to the analysis. Estimated beta regression coefficients, presented with corresponding 95% confidence interval (CI), were calculated as per standard deviation to compare the effects of different coagulation-related factors on TG parameters. Due to its explorative nature, a *p*-value threshold was not defined. However, to account for multiple statistical tests and minimize the risk of type 1 error, a Bonferroni corrected *p*-value (0.00036) was set for the results on the multiple linear regression analyses.

## Results

### Baseline characteristics of the study sample

Baseline characteristics of the reference group, arterial and venous disease groups are shown in Table 1. The majority of the individuals in the arterial subsample were males (63.3%), whereas there was a preponderance of females in the reference group (60.5%) and the venous disease group (63.6%). The mean age in the reference group was 49.3 years and the mean age of the study population in the arterial and venous disease groups was 63.8 years and 61.3 years, respectively. In both the arterial and venous disease groups, hypertension (arterial disease group: 72.5%, venous disease group: 59.0%) was the most prevalent traditional CVRF, followed by family history of MI/stroke (arterial disease group: 43.6%, venous disease group: 43.6%). Of the cardiovascular diseases, CAD was the most prevalent with 46.5% of the study subjects in the arterial disease group. In the venous disease group, 99.0% of the individuals had a history of DVT and 5.7% of the individuals had a history of PE. Of the arterial vascular diseases, PAD was predominant with 26.6% of the study subjects in the venous disease group. Anti-coagulant therapy was most common in the arterial disease group (61.0%), followed by the venous disease group (35.4%) and the reference group (1.8%). While individuals from the reference group were most often taking oral contraceptive therapy (12.5%), individuals in the arterial disease group were most often using hormonal replacement therapy (11.3%).

**Table 1:** Study sample characteristics

	Reference group *n* = 1402	Arterial Disease group *n* = 502	Venous Disease group *n* = 195
Sex (females)	60.5% (848)	36.7% (184)	63.6% (124)
Age(years)	49.3 ± 9.8	63.8 ± 8.5	61.3 ± 10.1
BMI (kg/m^2^)	24.2 (22.2/26.3)	28.8 (25.7/32.2)	28.6 (25.5/31.5)
**Cardiovascular risk factors**			
Diabetes	0% (0)	20.1% (101)	9.7% (19)
Obesity	0% (0)	41.2% (207)	36.9% (72)
Smoking	22.5% (316)	16.2% (81)	11.3% (22)
Arterial hypertension	0% (0)	72.5% (364)	59.0% (115)
Dyslipidemia	0% (0)	40.1% (201)	30.8% (60)
Family history of MI/Stroke	31.1% (436)	43.6% (219)	43.6% (85)
**Comorbidities**			
CAD	0% (0)	46.5% (217)	9.1% (17)
MI	0% (0)	30.4% (151)	5.2% (10)
Stroke	0% (0)	18.5% (92)	6.2% (12)
AF	0% (0)	9.2% (45)	6.2% (12)
PAD	0% (0)	40.2% (200)	26.6% (51)
CHF	0% (0)	6.2% (31)	6.7% (13)
DVT	0% (0)	14.3% (71)	99.0% (193)
PE	0% (0)	1.6% (8)	5.7% (11)
**Therapy**			
Anti-coagulant agents^a^	1.8% (25)	61.0% (305)	35.4% (69)
Oral contraceptive therapy	12.5% (174)	6.4% (32)	9.7% (19)
Hormonal replacement therapy	5.5% (77)	5.6% (28)	11.3% (22)

Abbreviations: *BMI* body mass index, *MI* myocardial infarction, *AF* atrial fibrillation, *PAD* peripheral artery disease, *CAD* coronary artery disease, *CHF* congestive heart failure, *DVT* deep venous thrombosis, *PE* pulmonary embolism

^a^ATC codes: B01AA (vitamin K antagonists), B01AB (heparin group), B01AE (direct thrombin inhibitors), B01AF (direct factor Xa inibitors), B01AX (other antithrombotic agents)

### Levels of coagulation factors and inhibitors

Levels of coagulation factors and inhibitors in the reference group, arterial and venous disease group are shown in Table 2. Most notably, the lag time was significantly prolonged in individuals with a history of arterial vascular disease or venous thrombosis in comparison to the cardiovascular healthy individuals. In addition, the ETP from the arterial disease group was lower compared to the reference group. The activity level of factors II, X and antithrombin were lower in the arterial and venous disease groups compared to the reference group. Differently, activity levels of factors VIII and XI, vWF activity and fibrinogen concentration were higher in both arterial and venous disease groups compared to reference group. The individuals from the arterial disease group compared to the control subjects showed additionally higher activity levels of factor IX and Protein S and slightly lower activity of factor XII.

**Table 2:** Parameters of thrombin generation and levels of natural coagulation and anti-coagulant factors in the reference, arterial and venous subsample

Variable	Group				
	Reference group (*n* = 1402) Mean ± SD	Arterial Disease group (*n* = 502) Mean ± SD	*p*-value arterial disease vs. reference	Venous Disease group (*n* = 195) Mean ± SD	*p*-value venous disease vs. reference
Lag Time [min]	4.94 ± 1.03	6.56 ± 3.71	**< 0.0001**	6.50 ± 3.95	**< 0.0001**
ETP [nM.min]	1105.0 ± 237.4	1045.0 ± 360.8	**0.00056**	1048.5 ± 423.3	0.070
Peak Height [nM]	118.37 ± 54.81	113.09 ± 55.36	0.067	113.50 ± 60.32	0.29
Factor II [%]	116.9 ± 17.6	108.7 ± 28.9	**< 0.0001**	104.8 ± 32.7	**< 0.0001**
Factor V [%]	116.6 ± 18.5	118.9 ± 19.9	**0.024**	118.4 ± 22.0	0.29
Factor VII [%]	111.1 ± 21.6	108.3 ± 31.2	0.056	107.2 ± 34.8	0.12
Factor VIII [%]	115.3 ± 33.1	136.3 ± 40.2	**< 0.0001**	140.1 ± 46.5	**< 0.0001**
Factor IX [%]	106.3 ± 14.4	110.9 ± 21.7	**< 0.0001**	107.4 ± 24.3	0.53
Factor X [%]	113.10 ± 19.09	105.59 ± 33.53	**< 0.0001**	103.87 ± 38.95	**0.0013**
Factor XI [%]	106.8 ± 18.2	110.2 ± 19.8	**0.00090**	112.6 ± 19.4	**< 0.0001**
Factor XII [%]	105.6 ± 24.3	102.1 ± 24.9	**0.0071**	105.5 ± 24.7	0.98
vWF [%]	103.1 ± 35.5	127.7 ± 45.0	**< 0.0001**	127.7 ± 48.3	**< 0.0001**
Protein C [%]	113.3 ± 17.2	111.3 ± 26.1	0.097	110.2 ± 28.3	0.13
Protein S [%]	95.1 ± 16.4	100.3 ± 24.1	**< 0.0001**	93.4 ± 24.7	0.34
Antithrombin [%]	102.8 ± 9.8	98.1 ± 11.9	**< 0.0001**	100.8 ± 10.7	**0.014**
TFPI Activity [U/mL]	1.57 ± 0.53	1.63 ± 0.57	0.040	1.64 ± 0.52	0.084
Fibrinogen [mg/dL]	331 ± 66	395 ± 95	**< 0.0001**	391 ± 95	**< 0.0001**

Presented are thrombin generation parameters and coagulation factors in the reference, arterial disease and venous disease subsample

Abbreviations: *SD* standard deviation, *vWF* Von Willebrand Factor, *TFPI* Tissue Factor Pathway Inhibitor

### Determinants of thrombin generation

**Fig. 1:**
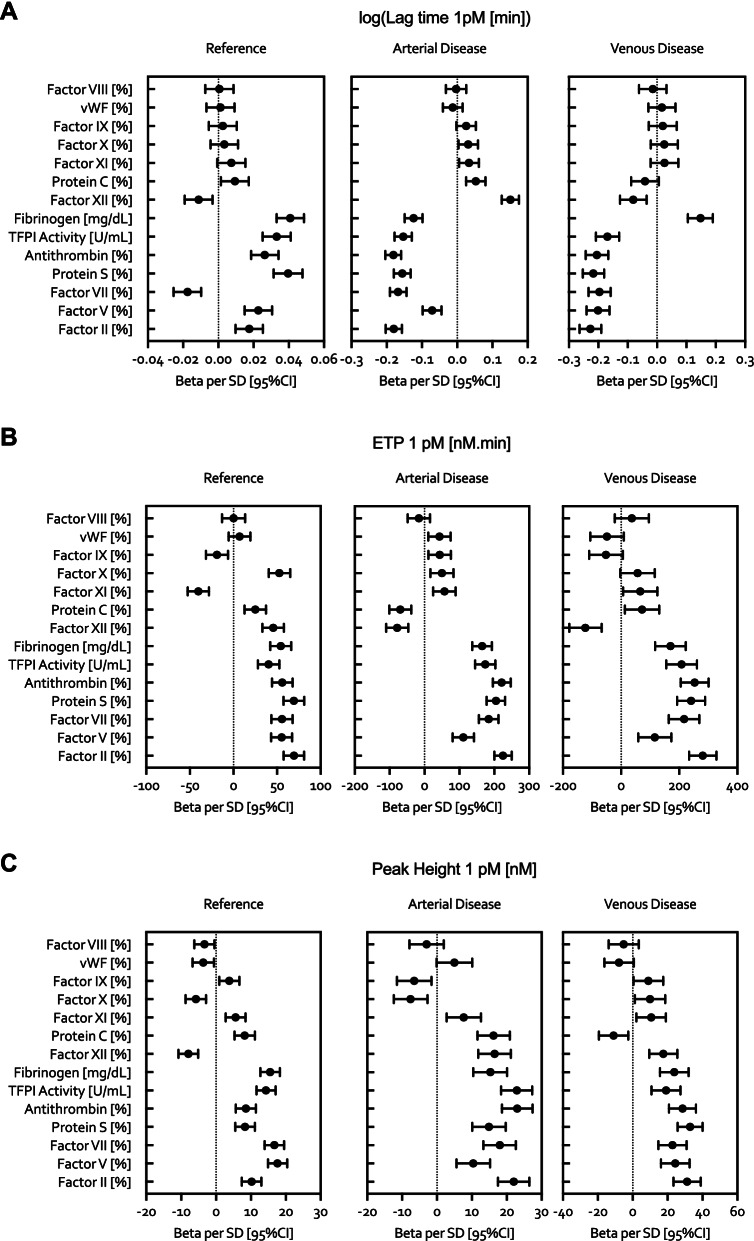
Multivariate analysis for relationships between coagulation factors and the TG assay in the reference group, arterial and venous disease group. The relationships beween coagulation factors and the lag time (panel **A**), ETP (panel **B**) and peak heigh (panel **C**) of thrombin generation are provided for the reference group (left panels), the arterial disease (middle panels) and venous disease (right panels) groups. The multiple linear regression models were adjusted for age, sex and medication. Abbreviations: vWF, Von Willebrand Factor; TFPI, Tissue Factor Pathway Inhibitor. * ATC codes: B01AA (vitamin K antagonists), B01AB (heparin group), B01AE (direct thrombin inhibitors), B01AF (direct factor Xa inhibitors), B01AX (other antithrombotic agents)

The multivariate analysis for relationships between coagulation factors and the TG assay in the reference group, arterial and venous disease group is presented in Fig. 1A-C. Presented in supplemental material Table 1A-C are beta per standard deviation SD, meaning that one SD change of the predictor (coagulation factors) leads to beta change in dependent variable (TG parameter). The lag time in the arterial and venous disease group was strongly and negatively associated with coagulation factors II, V and VII and with the natural anti-coagulants protein S, antithrombin and TFPI activity. Fibrinogen was negatively associated with the lag time in the arterial disease group and positively associated in the venous disease group. Differently, factor XII was positively associated with the lag time in the arterial disease group and negatively associated in the venous disease group. In general, the effect size of the reported biochemical determinants was 10 times higher for the arterial and venous disease groups compared to the reference group (e.g. factor II, beta estimate median: arterial: − 0.18 vs venous − 0.23 vs reference 0.017). In addition, the direction of the associations for the reference group was positive for all reported variables, except for factor VII that was negatively associated.

The ETP was strongly positively determined by factor II, VIII, X and IX in the reference group as well as the arterial and venous disease groups (Fig. 1A-C). In addition, antithrombin was a negative determinant for the ETP in the reference group, though no association was observed for antithrombin in the arterial and venous disease groups. VWF was a negative determinant for the ETP in both the arterial and venous disease group, whereas VWF was positively associated with the ETP in the reference group. Moreover, the effect size of the reported relationships was nearly 4 times higher for the arterial and venous disease groups in comparison to the reference group (e.g. factor VIII, beta estimate median: arterial 184 vs venous 217 vs reference 55.6).

There was a positive association between factor VIII, IX, II, X and the peak height in the reference group, arterial and venous disease group. (Fig. 1A-C) Protein S was a negative determinant of the peak height in the reference group, whereas it was positively associated with the peak height in the arterial and venous disease group. In addition, factor IX was a negative determinant for the peak height in the reference group, though no association was found in the arterial and venous disease group. In general, the effect sizes for the reported biochemical determinants of the peak height were similar in all groups, with the exception of factor VII that had lesser effect in the arterial disease group compared to the reference group and venous disease group (beta estimate: arterial: 10.4 vs venous 24.4 reference 17.6).

## Discussion

This is the first large scale population-based study that has explored the relationships between coagulation factors and the TG parameters in individuals with a history of arterial vascular disease or venous thrombotic disease, as compared to cardiovascular healthy individuals. The main findings from our study show important distinct differences for the biochemical determinants between cardiovascular healthy individuals and those with a background of an arterial or venous disease. Whereas lag time was mainly negatively associated with the procoagulant and anti-coagulant factors in the plasma, meaning higher factor levels result in a shorter lag time, the same associations were positive for the healthy individuals. Furthermore, the effect size for the biochemical parameters determining the lag time was about 10 times higher for the arterial and venous disease than for the reference group.

Dielis et al. previously investigated the coagulation factors as determinants of the TG parameters at 1 pM TF (comparable to the applied PPP Reagent Low) and 13.6 pM TF in the absence or presence of thrombomodulin or in the absence or presence of activated protein C in a sample of healthy adults [21]. TFPI activity, protein S and fibrinogen were the strongest positive determinants of the lag time. Similarly, the results of the present study showed that TFPI activity, protein S and fibrinogen are strong positive determinants of the lag time in the control individuals. Fibrinogen was also positively associated with the lag time from the venous disease group. A possible explanation for the paradoxical association between fibrinogen and lag time may be the anti-coagulant properties of fibrinogen by inhibiting the binding with thrombin directly as well as through accelerating the activation of plasminogen into plasmin by tissue plasminogen activator [22]. Interestingly, for the arterial disease individuals, higher fibrinogen concentration was associated with shorter lag time. These contrasting results raise the possibility of differential effects of fibrinogen on the initiation phase of the TG process in diseases affecting different vascular beds. Factor VII was the unique coagulation factor that shared the same direction of association with the lag time for control subjects and disease individuals. Factor VII is well known to play an important role in the initiation phase of the coagulation cascade by formation of the factor VIIa/TF complex that promotes the generation of the prothrombinase complex (factor Xa/factor Va) and ultimately leads to TG amplification [23]. Higher factor VII activity level and shorter lag time, shared by both control and disease individuals, confirms the role of factor VII in the ambient coagulation cascade reaction.

Furthermore, Dielis and colleagues reported fibrinogen and factor XII as positive determinants for the ETP, which we confirmed in the present study [21]. As expected and as previously reported, antithrombin, a potent anti-coagulant, was negatively associated with the ETP. In general, the present analysis demonstrated that the direction of associations with coagulation factors and ETP were similar in the reference group, arterial and venous disease group.

The analysis of the levels of natural coagulation and anti-coagulant factors showed that factor II, VIII, X and XI were significantly increased in the subjects with an arterial or venous disease background in comparison to the healthy individuals, which is in accordance with previous reports [9, 24–29]. This finding illustrates a “hypercoagulable” state in these subjects and may explain the fourfold increased effect size of the associations with the reported coagulation factors and the ETP in arterial and venous disease groups compared to the reference group. This is further supported by evidence from previous TG assay studies demonstrating its potential to expose hypercoagulability in plasma from patients with arterial and venous thrombosis [30].

In contrast to the reference group, protein S, a natural anti-coagulant, was a positive determinant for the peak height in individuals with a history of arterial or venous thrombotic disease. Our analysis confirms increased levels of coagulation factors in patients with an arterial or venous thrombotic disease background, which could potentially result in excessive activation of the activated protein C pathway to which protein S is a supporting cofactor. Therefore, as demonstrated by the analysis from the arterial disease group, levels of protein S may be elevated. However, the net effect of these pathological mechanism remains an increased thrombin generation which translates to the increased peak height. The effect sizes of the associations with the peak height were similar for healthy individuals and individuals with an arterial or venous thrombotic disease background.

Comparing the determinants of thrombin generation from the arterial disease group with patients suffering from acute myocardial infarction [10], reveals an opposite negative association between fibrinogen levels with the ETP and peak height at the acute phase. To what extend the associations in the acute phase are comparable to those after the event, remains to be elucidated.

Limitations to the study were: The TG was measured in PPP after one-step centrifugation of whole blood (10 minutes at 2000 x *g*), in contrast to standard recommendations (two-step centrifugation; 2000 x *g* for 5 minutes, 10,000 x *g* for 10 minutes), which may affect the TG results. The history of arterial or venous disease was self-reported by the participants. There was no data available for analysis on the time from the initial diagnosis of the arterial and/or venous event to study enrolment. Therefore, we were not able to investigate if different duration of disease has different impact on the coagulation and TG profile.

However, this study has important strengths, including the standardized clinical investigation of the participants’ present cardiovascular profile and the comprehensive laboratory investigation of coagulation and anti-coagulant factors.

In conclusion, this large-scale analysis shows that the individual coagulation factors more strongly affect TG parameters in individuals with a history of arterial or venous thrombosis as compared to cardiovascular healthy individuals. This illustrates the different effect size contribution of the coagulation factors to the hypercoagulable state of individuals at risk for a cardiovascular event and suggests that the coagulome might be tuned to a “hypersensitive” state increasing the risk for recurrence. Overall, the important finding of altered determinants of thrombin generation shows that in patients with a history of cardiovascular disease levels of coagulation factors should be taken into account. It also provides further rationale for the observed benefits of anti-coagulant therapy in patients with cardiovascular disease at risk of atherothrombosis.

## Supplementary Information

**Additional file 1.**

## Data Availability

The dataset used and/or analysed during the current study are available from the corresponding author on reasonable request.
