# Supplementary material for: Relationships between coagulation factors and thrombin generation in a general population with arterial and venous disease background
Source: Thromb J. 2022 Jun 8;20:32. doi: 10.1186/s12959-022-00392-0 (PMC9175351; doi:10.1186/s12959-022-00392-0)
Supplement: Supplementary file 1 — Additional file 1. [file 12959_2022_392_MOESM1_ESM.docx]

**SUPPLEMENTAL MATERIAL**

Biochemical determinants of thrombin generation in a general population with arterial and venous disease background

Pauline C.S. van Paridon^1,2^, Marina Panova-Noeva^2,8^, Rene van Oerle^1^, Andreas Schulz^3^, Jürgen H. Prochaska^2,3,8^, Natalie Arnold^3^, Irene Schmidtmann^4^, Manfred Beutel^5^, Norbert Pfeiffer^6^, Thomas Münzel^7,8^, Karl J. Lackner^9,8^, Hugo ten Cate^1,2^, Philipp S. Wild^2,3,8^ and Henri M.H. Spronk^1^

^1^ Laboratory for Clinical Thrombosis and Hemostasis, Department of Internal Medicine, Cardiovascular Research Institute Maastricht (CARIM), Maastricht University Medical Center, Maastricht, The Netherlands

^2^ Center for Thrombosis and Hemostasis (CTH), University Medical Center of the Johannes Gutenberg-University Mainz, Germany

^3^ Preventive Cardiology and Preventive Medicine, Center for Cardiology, University Medical Center of the Johannes Gutenberg-University Mainz, Germany

^4^ Institute of Medical Biostatistics, Epidemiology and Informatics, University Medical Center of the Johannes Gutenberg-University Mainz, Germany

^5^ Department of Psychosomatic Medicine and Psychotherapy, University Medical Center of the Johannes Gutenberg-University Mainz, Germany

^6^ Department of Ophthalmology, University Medical Center of the Johannes Gutenberg-University Mainz, Germany

^7^ Center for Cardiology I, University Medical Center of the Johannes Gutenberg-University Mainz, Germany

^8^ DZHK (German Center for Cardiovascular Research), Partner Site RhineMain, Mainz, Germany

^9^ Institute for Clinical Chemistry and Laboratory Medicine, University Medical Center of the Johannes Gutenberg-University Mainz, Germany

Part A. Supplemental methods

*Definition of classical cardiovascular risk factors*

Diabetes mellitus and dyslipidemia were defined as individuals with a definite diagnosis of by a physician. Additional definition of diabetes was a blood glucose level of ≥126mg/dl in the baseline examination after an overnight fast of at least 8 hours or a blood glucose level of ≥200mg/dl in the baseline examination after a fasting period <8 hours. Dyslipidemia was additionally defined as a LDL/HDL-ratio of >3.5. Hypertension was diagnosed, if antihypertensive drugs are taken, or a mean systolic blood pressure of ≥140mmHg or a mean diastolic blood pressure of ≥90mmHg (in the 2nd and 3rd standardized measurement after 8 and 11 minutes of rest). Smoking was classified into non-smokers (never smokers and former smokers) and smokers (occasional smoker, i.e. <1 cigarette/day, and smoker, i.e. ≥1 cigarette/day). Obesity defined as a body-mass index ≥ 30 kg/m². Self-reported coronary artery disease (CAD), myocardial infarction (MI), heart failure (HF), stroke, deep vein thrombosis (DVT), pulmonary embolism (PE) and peripheral arterial disease (PAD) indicated personal history of cardiovascular disease. A positive family history was defined as history of myocardial infarction or stroke in a female first-degree relative ≤65 years or a male first-degree relative ≤60 years.

*Categorization of medication*

Medications were classified according to the Anatomical Therapeutic Chemical (ATC) classification system. The following medication groups were selected for analysis: anticoagulant agents (B01), sex hormones and modulators of the genital system (G03). For the use of oral contraceptives and/or hormone replacement therapy, both ATC-code and self-reported information were used.

| **Supplemental table 1A-C.** Biochemical determinants of the TG parameters in the reference subsample and arterial and venous subsample | | | | | | |
| --- | --- | --- | --- | --- | --- | --- |
| **1A. Lag time** | | | | | | |
| **Group:** | **Reference** | | **Arterial disease** | | **Venous disease** | |
| **log(Lag time 1pM [min])** | **Beta per SD (95% CI)** | **p value** | **Beta per SD (95% CI)** | **p value** | **Beta per SD (95% CI)** | **p value** |
| Factor II [%] | 0.0175(0.00972/0.0253 ) | <0.0001 | -0.18 (-0.203/-0.157 ) | <0.0001 | -0.227(-0.264/-0.190 ) | <0.0001 |
| Factor V [%] | 0.0227 (0.0149/0.0305 ) | <0.0001 | -0.0712 (-0.0981/-0.0444 ) | <0.0001 | -0.201 (-0.240/-0.162 ) | <0.0001 |
| Factor VII [%] | -0.0175 (-0.0254/-0.00972 ) | <0.0001 | -0.168 (-0.191/-0.144 ) | <0.0001 | -0.196 (-0.233/-0.158 ) | <0.0001 |
| Protein S [%] | 0.0396 (0.0314/0.0477 ) | <0.0001 | -0.156 (-0.180/-0.132 ) | <0.0001 | -0.217 (-0.253/-0.180 ) | <0.0001 |
| Antithrombin [%] | 0.0263 (0.0186/0.0341 ) | <0.0001 | -0.181 (-0.204/-0.159 ) | <0.0001 | -0.205 (-0.243/-0.166 ) | <0.0001 |
| TFPI Activity [U/mL] | 0.0331 (0.0251/0.0411 ) | <0.0001 | -0.153 (-0.178/-0.129 ) | <0.0001 | -0.169 (-0.208/-0.129 ) | <0.0001 |
| Fibrinogen [mg/dL] | 0.0408 (0.0330/0.0486 ) | <0.0001 | -0.124 (-0.149/-0.0986 ) | <0.0001 | 0.148 (0.105/0.190 ) | <0.0001 |
| Factor XII [%] | -0.0112 (-0.0191/-0.00332 ) | 0.0053 | 0.151 (0.126/0.175 ) | <0.0001 | -0.081 (-0.126/-0.0356 ) | 0.00047 |
| Protein C [%] | 0.0094 (0.00148/0.0173 ) | 0.02 | 0.0527 (0.0250/0.0804 ) | 0.00019 | -0.041 (-0.0883/0.00620 ) | 0.089 |
| Factor XI [%] | 0.00737 (-0.000621/0.0154 ) | 0.071 | 0.0333 (0.00536/0.0612 ) | 0.019 | 0.0254 (-0.0218/0.0726 ) | 0.29 |
| Factor X [%] | 0.00335 (-0.00445/0.0112 ) | 0.4 | 0.0312 (0.00340/0.0591 ) | 0.028 | 0.0246 (-0.0214/0.0706 ) | 0.29 |
| Factor IX [%] | 0.00253 (-0.00542/0.0105 ) | 0.53 | 0.0255 (-0.00218/0.0531 ) | 0.071 | 0.0195 (-0.0280/0.0670 ) | 0.42 |
| vWF [%] | 0.00121 (-0.00681/0.00923 ) | 0.77 | -0.0127 (-0.0405/0.0152 ) | 0.37 | 0.0166 (-0.0294/0.0627 ) | 0.48 |
| Factor VIII [%] | 0.000547 (-0.00746/0.00855 ) | 0.89 | -0.00312 (-0.0320/0.0257 ) | 0.83 | -0.0144 (-0.0612/0.0324 ) | 0.55 |

| **1B. ETP** | | | | | | |
| --- | --- | --- | --- | --- | --- | --- |
| Subsample: | **Reference** | | **Arterial disease** | | **Venous disease** | |
| **ETP 1pM [nM.min]** | **Beta per SD (95% CI)** | **p value** | **Beta per SD (95% CI)** | **p value** | **Beta per SD (95% CI)** | **p value** |
| Factor II [%] | 69.3 (57.5/81.0 ) | <0.0001 | 225 (200/250) | <0.0001 | 281 (234/328) | <0.0001 |
| Factor VII [%] | 55.2 (43.2/67.2 ) | <0.0001 | 111 (80.8/142) | <0.0001 | 116 (59.2/173) | <0.0001 |
| Factor VIII [%] | 55.6 (43.4/67.8 ) | <0.0001 | 184 (156/212) | <0.0001 | 217 (164/269) | <0.0001 |
| Factor IX [%] | 69.2 (57.3/81.1 ) | <0.0001 | 205 (178/231) | <0.0001 | 241 (193/289) | <0.0001 |
| Factor X [%] | 55.9 (44.1/67.8 ) | <0.0001 | 221 (196/247) | <0.0001 | 253 (205/301) | <0.0001 |
| Factor XI [%] | 40.2 (27.9/52.6 ) | <0.0001 | 174 (145/203) | <0.0001 | 208 (155/261) | <0.0001 |
| Factor XII [%] | 54.2 (42.1/66.2 ) | <0.0001 | 165 (137/193) | <0.0001 | 170 (117/222) | <0.0001 |
| vWF [%] | 45.5 (33.1/57.8 ) | <0.0001 | -78.6 (-110/-46.8 ) | <0.0001 | -123 (-178/-67.1 ) | <0.0001 |
| Protein C [%] | 25 (12.6/37.3 ) | <0.0001 | -69.5 (-101/-38.2 ) | <0.0001 | 71.9 (12.9/131) | 0.017 |
| Antithrombin [%] | -40.4 (-52.5/-28.3 ) | <0.0001 | 57 (24.8/89.2 ) | 0.00052 | 66 (6.74/125) | 0.029 |
| Fibrinogen [mg/dL] | 52.6 (40.3/65.0 ) | <0.0001 | 49.9 (16.8/83.0 ) | 0.0031 | 56.6 (-2.58/116) | 0.061 |
| TFPI Activity [U/mL] | -19 (-31.7/ -6.32 ) | 0.0033 | 43.4 (11.0/75.7 ) | 0.0085 | -52.2 (-110/5.90 ) | 0.078 |
| Factor V [%] | 6.96 (-5.47/19.4 ) | 0.27 | 42.8 (10.8/74.9 ) | 0.0089 | -48.7 (-106/9.06 ) | 0.098 |
| Protein S [%] | 0.0931 (-13.1/13.3 ) | 0.99 | -16.2 (-48.3/15.9 ) | 0.32 | 37.1 (-21.7/95.8 ) | 0.22 |

| **1C. Peak height** | | | | | | |
| --- | --- | --- | --- | --- | --- | --- |
| **Subsample:** | **Reference** | | **Arterial disease** | | **Venous disease** | |
| **Peak height 1pM [nM]** | **Beta per SD (95% CI)** | **p value** | **Beta per SD (95% CI)** | **p value** | **Beta (per SD) (95% CI)** | **p value** |
| Factor II [%] | 10.2 (7.38/13.0 ) | <0.0001 | 22 (17.5/26.5 ) | <0.0001 | 31.2 (23.4/39.0 ) | <0.0001 |
| Factor VII [%] | 17.6 (14.9/20.4 ) | <0.0001 | 10.4 (5.60/15.2 ) | <0.0001 | 24.4 (16.2/32.6 ) | <0.0001 |
| Factor VIII [%] | 16.7 (13.9/19.5 ) | <0.0001 | 18 (13.3/22.6 ) | <0.0001 | 22.8 (14.7/30.9 ) | <0.0001 |
| Factor IX [%] | 8.28 (5.48/11.1 ) | <0.0001 | 14.9 (10.1/19.7 ) | <0.0001 | 32.9 (25.7/40.1 ) | <0.0001 |
| Factor X [%] | 8.53 (5.66/11.4 ) | <0.0001 | 23 (18.6/27.4 ) | <0.0001 | 28.6 (20.8/36.3 ) | <0.0001 |
| vWF [%] | 14.3 (11.6/17.1 ) | <0.0001 | 22.9 (18.4/27.3 ) | <0.0001 | 19.1 (10.8/27.4 ) | <0.0001 |
| Protein C [%] | 15.5 (12.7/18.3 ) | <0.0001 | 15.3 (10.4/20.1 ) | <0.0001 | 23.8 (15.6/32.0 ) | <0.0001 |
| Protein S [%] | -7.93 (-10.8/-5.11 ) | <0.0001 | 16.5 (11.8/21.2 ) | <0.0001 | 17.5 (9.46/25.6 ) | <0.0001 |
| TFPI Activity [U/mL] | 8.18 (5.27/11.1 ) | <0.0001 | 16.2 (11.6/20.9 ) | <0.0001 | -11 (-19.4/-2.55 ) | 0.011 |
| Factor V [%] | 5.6 (2.77/8.44 ) | 0.00011 | 7.66 (2.77/12.6 ) | 0.0021 | 10.6 (2.08/19.0 ) | 0.015 |
| Factor XI [%] | -5.8 (-8.73/-2.87 ) | 0.00011 | -7.58 (-12.4/-2.75 ) | 0.0021 | 9.92 (1.29/18.6 ) | 0.024 |
| Factor XII [%] | 3.79 (0.916/6.67 ) | 0.0098 | -6.55 (-11.5/-1.60 ) | 0.0094 | 8.98 (0.470/17.5 ) | 0.039 |
| Antithrombin [%] | -3.7 (-6.75/-0.652 ) | 0.017 | 4.99(-0.101/10.1 ) | 0.055 | -7.86 (-16.3/0.545 ) | 0.067 |
| Fibrinogen [mg/dL] | -3.35 (-6.23/-0.476 ) | 0.022 | -2.99 (-7.91/1.94 ) | 0.23 | -5.19 (-13.8/3.42 ) | 0.24 |
| The multiple linear regression models were adjusted for age, sex and medication. Abbreviations: vWF, Von Willebrand Factor; TFPI, Tissue Factor Pathway Inhibitor. * ATC codes: B01AA (vitamin K antagonists), B01AB (heparin group), B01AE (direct thrombin inhibitors), B01AF (direct factor Xa inhibitors), B01AX (other antithrombotic agents). | | | | | | |
